# Molecular Characterization of *Clostridium difficil*e Isolates in China From 2010 to 2015

**DOI:** 10.3389/fmicb.2018.00845

**Published:** 2018-04-30

**Authors:** Xiao-shu Liu, Wen-ge Li, Wen-zhu Zhang, Yuan Wu, Jin-xing Lu

**Affiliations:** State Key Laboratory for Infectious Disease Prevention and Control, Collaborative Innovation Center for Diagnosis and Treatment of Infectious Diseases, National Institute for Communicable Disease Control and Prevention, Chinese Center for Disease Control and Prevention, Beijing, China

**Keywords:** *Clostridium difficile*, MLST, toxin genes profile, PCR-ribotyping, molecular characterization

## Abstract

*Clostridium difficile* infection (CDI) has become a worldwide public health problem causing high mortality and a large disease burden. Molecular typing and analysis is important for surveillance and infection control of CDI. However, molecular characterization of *C*. *difficile* across China is extremely rare. Here, we report on the toxin profiles, molecular subtyping with multilocus sequence typing (MLST) and PCR ribotyping, and epidemiological characteristics of 199 C. difficile isolates collected between 2010 through 2015 from 13 participating centers across China. We identified 35 STs and 27 ribotypes (RTs) among the 199 C. difficile isolates: ST35 (15.58%), ST3 (15.08%), ST37 (12.06%), and RT017 (14.07%), RT001 (12.06%), RT012 (11.56%) are the most prevalent. One isolate with ST1 and 8 isolates with ST 11 were identified. We identified a new ST in this study, denoted ST332. The toxin profile *tcdA*^+^*tcdB*^+^*tcdC*^+^*tcdR*^+^*tcdE*^+^CDT^-^ (65.83%) was the predominant profile. Furthermore, 11 isolates with positive binary toxin genes were discovered. According to the PCR ribotyping, one isolate with RT 027, and 6 isolates with RT 078 were confirmed. The epidemiological characteristics of *C. difficile* in China shows geographical differences, and both the toxin profile and molecular types exhibit great diversity across the different areas.

## Introduction

*Clostridium difficile* is considered to be the leading cause of antimicrobial-associated diarrhea and has been observed in older inpatients (Rupnik et al., 2009). *C. difficile* infection (CDI) caused by toxigenic strains show a wide range of clinical symptoms from diarrhea to pseudomembranous colitis (Dingle et al., 2011), and may even result in tissue damage or severe dehydration (Lessa et al., 2012; Rineh et al., 2014). CDI has become a worldwide public health problem (Lessa et al., 2012; Rineh et al., 2014; Sun and Hirota, 2015). The major pathogenic mechanism of *C. difficile* is the production of enterotoxin A and cytotoxin B, encoded by *tcdA* and *tcdB* genes, which are co-located in a 19.6 kb region of the chromosome named as Paloc with other regulatory genes (Freeman et al., 2010; Knight et al., 2015). In addition to toxins A and B, some *C.* *difficile* isolates can produce binary toxin, encoded by *cdtA* and *cdtB* genes, and it’s exact role in the pathogenesis of CDI is unknown (Voth and Ballard, 2005). Molecular methods such as PCR ribotyping, pulsed field gel electrophoresis (PFGE), multilocus variable number tandem repeat analysis (MLVA), multilocus sequence typing (MLST), restriction endonuclease analysis (REA), and amplified fragment length polymorphism (AFLP) have been applied to *C*. *difficile* isolates responsible for clinical infections (Knetsch et al., 2013). Recently, dramatically increased whole genome sequencing (WGS) help to define the architecture, diversity, conservation, and plasticity of the *C*. *difficile* genome, and provide a robust global phylogeny, evolution, and transmission of *C*. *difficile* (Knight et al., 2015). *C. difficile* BI/NAP1/027 and BK/NAP7/078 have been implicated in outbreaks and severe cases globally (Goorhuis et al., 2008; Kuijper et al., 2008; Gravel et al., 2009; Bauer et al., 2011). In recent years, an increasing number of studies of CDI in China have been performed within single hospitals or several hospitals in one place (Cheng et al., 2016; Jin et al., 2017). However, there is limited information on molecular characterization of *C*. *difficile* isolates in China, especially using longitudinal multicenter studies. In this study, we performed a molecular analysis of *C*. *difficile* isolates in China across several distinct geographic regions, spanning from 2010 to 2015. Here, the toxin profile and molecular characteristics of *C*. *difficile* are described. This study will help better understand the epidemiology of CDI in China and allow drafting strategies for control and prevention of CDI in China.

## Materials and Methods

### Study Samples and Case Definitions

This is a retrospective study including inpatients and outpatients with diarrhea of all ages from 13 tertiary hospitals across China from 2010 to 2015. There are three hospitals in Beijing, four hospitals in Shanghai, two hospitals in Shandong, and one hospital in Guangzhou, Hangzhou, Henan and Xi’an, respectively. Diarrhea was defined as with frequency over three times a day, accompanied by changes in fecal traits (Cohen et al., 2010). A total of 199 *C*. *difficile* strains was received or cultured at the laboratory in Chinese Centre for Disease Control and Prevention (China CDC) and characterized by molecular methods. Feces test is a convention inspection test in clinical for diarrhea patients. The patients knew and signed the written informed consent for sample collection and further test. Sample collection is coincided with the protocol of the participated hospitals and is approved by the Ethics Committees of China-Japan Friendship Hospital, Peking University First Hospital, Chinese PLA General Hospital, Huashan Hospital Affiliated to Fudan University, Shanghai East International Medical Center, Renjin Hospital Affiliated to School of Medicine of Shanghai Jiaotong University, Ruijin Hospital Affiliated to School of Medicine of Shanghai Jiaotong University, Affiliated Hospital of Taishan Medical University, The 5th People’s Hospital of Ji’nan, Nanfang Hospital Affiliated to Southern Medical University, Shao-yifu Hospital Affiliated to School of Medicine of Zhejiang University, Xijing Hospital Affiliated to the Fourth Military Medical University, and People’s Hospital of Ji Yuan.

In addition, 24 strains from the European Center for Disease Prevention and Control Brazier (ECDC-Brazier) collection and 30 strains from the American Type Culture Collection (ATCC) were used as standard reference strains for PCR-ribotyping in this study. The details of ATCC isolates are as follows: ATCC 9689 (RT001), ATCC 700057 (RT038), ATCC 43255 (RT087), ATCC 43594 (RT 005), ATCC 43601 (RT031), ATCC 17857 (RT001), ATCC17858 (RT 054), ATCC43593 (RT 060), ATCC43600 (RT014), ATCC43603 (RT085), ATCC43598 (RT017), ATCC BAA-1382 (RT012), BAA-2156 (RT118), BAA-1801 (RT010), BAA-1803 (RT027), BAA-1804 (RT053), BAA-1806 (RT220), BAA-1807 (RT140), BAA-1808 (RT020), BAA-1809 (RT009), BAA-1811 (RT057), BAA-1812 (RT024), BAA-1813 (RT002), BAA-1814 (RT251), BAA-1815 (RT076), BAA-1870 (RT027), BAA-1872 (RT207), BAA-1873 (RT053), BAA-1874 (RT002), BAA-1875 (RT078).

### Bacterial Culture, Identification and DNA Isolation

All fecal specimens were inoculated on selective cycloserine-cefoxitin-fructose agar plates (CCFA, Oxoid, United Kingdom) with 5% egg yolk after ethanol shock treatment and incubated in an anaerobic jar (Mart, NL) at 37°C for 48 h. After being vacuumed, an anaerobic atmosphere of 80% nitrogen, 10% hydrogen, and 10% carbon dioxide were injected. *C*. *difficile* colonies were identified on the basis of their typical morphology on agar plates and by Gram stain as well as the characteristic odor. Suspected colonies were further confirmed by API 20A (BioMerieux, France) for their biochemical characteristics and amplification of the GDH gene (Bauer et al., 2011), and the 16S rRNA gene (Lin et al., 2011).

DNA extraction was performed on all *C*. *difficile* isolates using a commercial DNA extraction kit (Tiangen, Beijing) based on the manufacturers’ instruction. The DNA samples were stored at -20°C for further use.

### Multilocus Sequence Typing (MLST)

Multilocus sequence typing was performed on all recovered isolates using the primers and methods developed by Griffiths et al. (2010). Seven housekeeping genes (*adk, atpA dxr, glyA, recA, sodA*, and *tpi*) were amplified and sequenced bi-directionally (**Table 1**). The complete allele sequences were analyzed using DNAStar and MEGA7 software and allele and ST assignments were performed using the *C. difficile* database at pubMLST.^1^ The neighbor joining (N-j) tree was constructed using the 100 *C. difficile* strains tested in this study, which was based on the seven combined housekeeping genes sequences using the MEGA7 software. A minimum spanning tree was created using BioNumerics version 5.10, according to the MLST data profile of 100 strains in this study and 99 isolates in our previous study (Yan et al., 2013).

**Table 1:** Primers used in this study.

Gene	Primer	Sequence (5′-3′)	Amplification Length(bp)	Reference
GDH	GDH-F	TTCCTAATTTAGCAGCAGCTTC	158	Bauer et al., 2011
	GDH-R	GTCTTGGATGGTTGATGAGTAC		
16srDNA	PS13	GGAGGCAGCAGTGGGGAATA	1,100	Lin et al., 2011
	PS14	TGACGGGCGGTGTGTACAAG		
adk	adkF	TTACTTGGACCTCCAGGTGC	501	Griffiths et al., 2010
	adkR	TTTCCACTTCCTAAGGCTGC		
atpA	atpAF	TGATGATTTAAGTAAACAAGCTG	555	Griffiths et al., 2010
	atpAR	AATCATGAGTGAAGTCTTCTCC		
dxr	dxrF	GCTACTTTCCATTCTATCTG	411	Griffiths et al., 2010
	dxrR	CCAACTCTTTGTGCTATAAA		
glyA	glyAF	ATAGCTGATGAGGTTGGAGC	516	Griffiths et al., 2010
	glyAR	TTCTAGCCTTAGATTCTTCATC		
recA	recAF	CAGTAATGAAATTGGGAGAAGC	564	Griffiths et al., 2010
	recAR	ATTCAGCTTGCTTAAATGGTG		
sodA	sodAF	CCAGTTGTCAATGTATTCATTTC	450	Griffiths et al., 2010
	sodAR	ATAACTTCATTTGCTTTTACACC		
tpi	tpiF	ATGAGAAAACCTATAATTGCAG	504	Griffiths et al., 2010
	tpiR	TTGAAGGTTTAACACTTCCACC		
tcdA	tcdA-F	AGATTCCTATATTTACATGACAATAT	36 (A+B+)	Lemee et al., 2004
	tcdA-R	GTATCAGGCATAAAGTAATATACTTT	110 (A-B+)	
tcdA	NK2	CCCAATAGAAGATTCAATATTAAGCT	251	Kato et al., 1991
	NK3	GGAAGAAAAGAACTTCTGGCACACTCAGGT		
tcdA rep	NK11	TGATGCTAATAATGAATCTAAAATGGTAAC	1,266	Kato et al., 1991
	NK9	CCACCAGCTGCAGCCATA		
tcdB	NK104	GTGTAGCAATGAAAGTCCAAGTTTACGC	203	Kato et al., 1991
	NK105	CACTTAGCTCTTTGATTGCTGCACCT		
tcdC	Tim2	GCACCTCATCACCATCTTCAA	345	Cohen et al., 2000
	Struppi2	TGAAGACCATGAGGAGGTCAT		
tcdC	C1	TTAATTAATTTTCTCTACAGCTATCC	718	Spigaglia and Mastrantonio, 2002
	C2	TCTAATAAAAGGGAGATTGTATTATG		
tcdR	Tim 3	AAAAGCGATGCTATTATAGTCAAA	300	Spigaglia and Mastrantonio, 2002
	Struppi3	CCTTATTAACAGCTTGTCTAGAT		
tcdE	Tim1	GTTTAAGTGCAATAAAAAGTCGTA	262	Cohen et al., 2000
	Struppi1	GGTAATCCACATAAGCACATATT		
cdtA	cdtApos	TGAACCTGGAAAAGGTGATG	375	Stubbs et al., 2000
	cdtArev	AGGATTATTTACTGGACCATTTG		
cdtB	cdtBpos	CTTAATGCAAGTAAATACTGAG	510	Stubbs et al., 2000
	cdt Brev	AACGGATCTCTTGCTTCAGTC		

### Toxin Gene Profiling and PCR-Ribotyping

The toxin genes profiling and PCR-ribotyping of 199 *C. difficile* strains were performed. 5% chelex-100(Bio-Rad) was used for DNA extraction. An alkaline environment (pH 7.5–8.0) is typically used for optimal interaction with magnesium. The boiling process and centrifugation processes (11,300 *g*) took 12 min. All isolates were screened by PCR for the presence of the toxin A (*tcdA*), toxin B (*tcdB*) genes, the binary toxin (*cdtA* and *cdtB*) genes, and the regulating genes of *tcdC, tcdR*, and *tcdE*. According to the literature (Kato et al., 1991, 1998; Cohen et al., 2000; Stubbs et al., 2000; Spigaglia and Mastrantonio, 2002; Lemee et al., 2004), the primers and PCR conditions for detection of toxins and regulation genes targets in PaLoc and CdtLoc from *C. difficile* were selected. A negative control (PCR grade water) and a positive control (RT027) were used for each PCR reaction. PCR reaction products were run on a QIAxcel capillary electrophoresis platform (QIAxcel Advanced DNA Screening Cartridge, QIAGEN). The primers used for PCR ribotyping have been described previously (O’Neill et al., 1996). The amplification conditions were as follows: 10 min at 95°C; 1 min at 94°C, 1 min at 58°C, and 2 min at 72°C for 25 cycles; 5 min at 72°C. PCR ribotyping reaction products were concentrated using a Qiagen Min-Elute PCR purification kit (QIAGEN) before being run on a QIAxcel capillary electrophoresis platform (QIAxcel Advanced DNA High Resolution Cartridge, QIAGEN). QIAxcel Advanced DNA High Resolution Cartridge (QIAGEN) was used to analyze of the ribotyping PCR purification products. Visualization of PCR products was performed with QIAxcel ScreenGel software (v1.3.0; QIAGEN). PCR ribotyping banding patterns were identified by comparison of banding patterns with a reference library consisting of a collection of 24 reference strains from ECDC, and a collection of 30 isolates from ATCC. Interpretation of the capillary electrophoresis data (PCR ribotyping banding patterns) was performed using the BioNumerics software package v.7.6 (QIAxcel Module). Isolates that could not be identified with the available reference library were designated with internal nomenclature.

## Results

**FIGURE 1:**
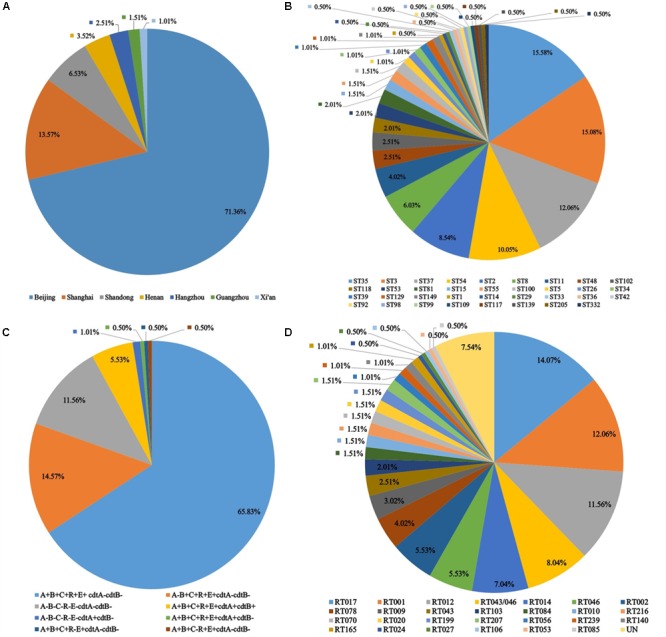
Isolation distribution, toxin profile and molecular types of 199 *C*. *difficile* isolates from 13 hospitals I China (2010–2015). **(A)** Distribution of strains by location of the origin hospitals. **(B)** Distribution of ST types. **(C)** Distribution of toxin gene profiles. **(D)** Distribution of RT types.

Of the 199 isolated *C. difficile* strains from 13 hospitals from 2010 to 2015, 142 strains were from Beijing, 7 strains from Henan, 27 strains from Shanghai, 5 strains from Hangzhou, 2 strains from Xi’an, 3 strains from Guangzhou, and 13 strains from Shandong (**Figure 1A**). A total of 170 patients were with intact demographical data, among which 67 were female, and 103 were male. The age of patients ranges from 30 days to 101 years. Five age groups were divided: 0–2 years (24 patients), 3–18 years (3 patients), 19–60 years (49 patients), 61–80 years (36 patients), and over than 80 years (58 patients).

### Multilocus Sequence Typing

**FIGURE 2:**
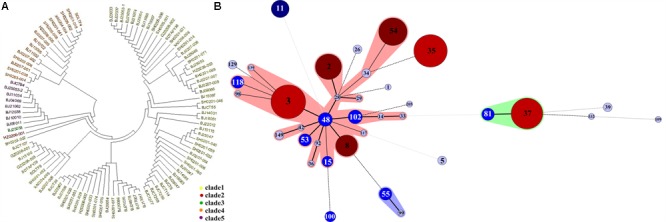
Phylogenetic analysis and population structure of *C*. *difficile* isolates from China according to MLST results. **(A)** The N-j tree of 100 isolates for MLST typing. Each color corresponds to clades (yellow, clade 1; red, clade 2; green, clade 3; orange, clade 4; purple, clade 5). These five clades were similar with reported population structure of *C*. *difficile* isolates around the world. Here, clade 1 were with heterogenecity and several sub-lineages were identified. **(B)** The minimum spanning tree of all 199 isolates in this study for MLST typing. Each circle corresponds to ST types, the number of which is indicated for the size of circles. The lines between circles indicate the similarity between profiles (bold, 5 alleles in common; normal, 4 alleles; dotted, ≤3 alleles). The shadow in the middle identified a clonal complex, in which ST48 right in the middle is the ancestor of other types.

**FIGURE 3:**
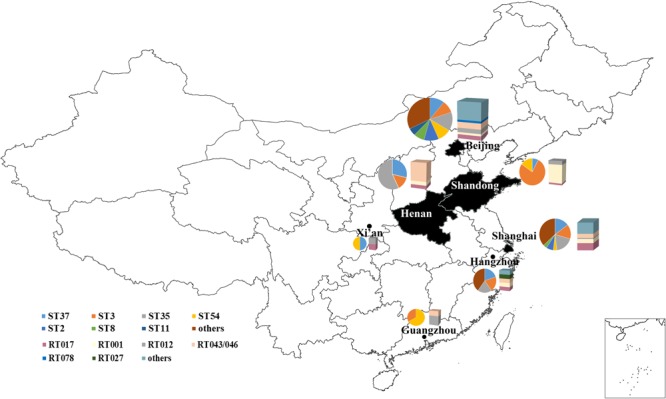
Geographical distribution and character of ST and RT types across China. The pie charts represent the ST distribution; the bar graphs represent the RT distribution. The predominant molecular types and composition were distinct among different locations.

Multilocus sequence typing results showed that the 199 *C. difficile* isolates formed 35 ST types, with a high degree of discrete characteristics (**Figure 1B**), within which 30 ST types were obtained from 100 *C. difficile* strains (S1). ST332 was a novel ST type. ST35 (15.58%), ST3 (15.08%), ST37 (12.06%), and ST54 (10.05%) were the most common ST types, as shown in **Figure 1B** and Supplemental Material. Notably, one ST1 from Hangzhou city and eight ST11 *C. difficile* isolates from Beijing were identified in this study. Most isolates belong to the large heterogeneous clade 1, which has subsequently evolutionary steps to MLST clades 4 and 5 (**Figure 2A**) (S1). There was only one isolate each in clade 2 and in clade 3 (**Figure 2A**). Considering our previous study, one more isolate was found in clade 3 (S1). A *C. difficile* cluster is obviously related to its genetic features but not by its geographic distribution or population groups. In the MLST scheme for the 199 *C. difficile* isolates, clade one (in the pink shadowed area) was the dominant groups (**Figure 2B**), and had differentiated distinctly from clades 3, 4 and 5 (**Figure 2B**). In clade 4, ST37 and ST81 were predominant and closely related with each other, but distinct from ST39, ST109 and ST332 (**Figure 2B**). In this study, clade 3 (ST5) and clade 5 (ST11) contained only one ST type (S1). For clade 1, several ST types, such as ST55, ST99, ST100 and others, were not included in the pink shadowed area (**Figure 2B**), although they were of the ST1 type, which further illustrates the significant heterogeneity of clade 1 (S1). The ST types of the strains have a high degree of discrete distribution characteristics (**Figure 3**), especially in Beijing and Shanghai. Almost all ST types were distributed across these two regions, and the ST composition was similar in these two areas. There were only two ST types identified in Xi’an (ST54 and ST37) and Guangzhou (ST54 and ST3). The primary ST types identified in Henan and Shandong were ST35 and ST3, respectively (**Figure 3**). In Hangzhou, the ST profile was similar to that in Beijing and Shanghai, but with fewer ST types (**Figure 3**). In addition, one ST1 isolate was identified here. The ST composition and distribution was different across the geographic regions. The 8 strains of ST11 were isolated from older hospitalized patients in the same hospital in Beijing.

### Toxin Gene Profile and PCR-Ribotyping

All the toxin genes (*tcdA, tcdB, cdtA-*, and *cdtB-*) and the regulatory genes (*tcdC, tcdR*, and *tcdE*) were screened in all 199 *C. difficile* isolates. Two pairs of primers were used for amplifying *tcdA* and *tcdC* genes, respectively. For *C. difficile* isolates in China, primers tcdA-F and tcdA-R displayed higher amplification efficiency than NK2 and NK3. Two pairs of primers of *tcd C*, Tim2 and Struppi2, C1 and C2, showed the same discriminatory power. There were 23 non-toxigenic strains (all the tested virulence genes and regulatory genes were negative) (S1). Except for the positive identification of *cdtA*, all tested genes were negative for isolates 14031 and 18051 (S1). This might be attributed to the variation of DNA, but whether this could be translated into functional protein needs further confirmation. The predominant toxin profile was *tcdA*^+^*tcdB^+^tcdC*^+^*tcdR^+^tcdE^+^cdtA^-^cdtB^-^*, accounting for 65.83% (**Figure 1C**). In addition, 11 isolates were detected with positive binary toxin (S1). The toxin profiles of all isolates were shown in **Figure 1C**, and details were summarized in the **Supplementary Table S1**.

A total of 27 RT types were identified from 182 isolates using the reference strains from the ECDC-Braizer collection and ATCC. The remaining 17 isolates were not classified using the standard RT library and then assigned a code in our laboratory (**Figure 1D**). The most frequent RT types distributed across China were RT017, RT001, and RT012 (**Figure 1D**). The composition of RT types was distinct among the geographic areas, which was similar to the distribution of ST (**Figure 3**). Notably, one isolate RT027 and 6 hypervirulent RT078 isolates were found in this study (**Figure 1D**). The RT types of all isolates were showed in **Supplementary Table S1**.

## Discussion

This is the first comprehensive and multicenter study of molecular characterization of *C*. *difficile* isolates in China across a large timeframe. The results revealed unique percentage of molecular types which is identified in previous studies in China (Tang et al., 2016; Tian et al., 2016). In our study, ST35, ST3, ST37, and ST54 were the most prevalent STs identified across China. In a systematic meta-analysis of CDI studies in China, ST37 and ST3 were the most prevalent types; hypervirulent strains, such as ST1 (BI/NAP1/027) and ST11 (RT 078), have only occurred sporadically to date (Tang et al., 2016). In mainland China, Tian et al. (2016) analyzed molecular characterization of *C*. *difficile* isolates from human subjects and the environment in North China. In their study, ST54 (29.2%), ST3 (25.7%), ST2 (9.7%), ST35 (10.6%), and ST37 (8.4%) were the dominant type (Tian et al., 2016). In our another study focusing community-acquired *C*. *difficile* infection in Yun Nan province in China (Liao et al., 2018), ST35, ST54, ST3 (RT001), and ST3 (RT009) were dominant types, however, no ST37 were found. Here, there were 30 ST3 isolates, comprising of 3 RTs as following: 1 isolate of UN, 6 of RT009, and the rest 23 isolates of RT001. It is illustrated that molecular epidemiological features of *C*. *difficile* might be affected by different population groups, geographic distribution, and habits and customs.

According to the RT results, RT017 accounted for the largest number, followed by RT001 and RT012. RT 017 with toxin gene profile A-B+ are widespread in Asia (Collins et al., 2013) and have caused epidemics worldwide (Kuijper et al., 2001). It has been reported that RT017 was identified as the predominant ribotype in previous studies in China (Huang et al., 2011; Gao et al., 2016). Recently, two distinct evenly split sub-lineages, SL1 (containing animal isolates) and SL2, of *C*. *difficile* RT017 were revealed through whole-genome sequencing (Cairns et al., 2017). In addition, 1 hypervirulent RT027 (ST1) and 6 RT078 (ST11) isolates were found in this study. RT027 has a 18 bp deletion in the *tcdC* gene, and produce an increased amount of toxins, causing greater severity and mortality. Six RT078 (ST11) of the 8 ST11 isolates displayed the same band with ECDC 078 and ATCC 1875 by capillary electrophoresis. Furthermore, all the 6 isolates were further confirmed as RT078, with a mutation point at position 184 and a Δ39-bp deletion. Our results corroborate previous findings that a single ST11 is associated with more than one PCR RT types, including RTs 033, 045, 066, 078, 126, and 193 (Knetsch et al., 2012). This suggests that PCR ribotyping is essential for subtyping of ST11 and ST1 (including RTs 016, 027, 036, and 176). The six RT078 isolates were all from old aged (>85 years) hospitalized patients in the same hospital in Beijing during November–December in 2015. There were three isolates (20086, ZR12, and ZR15) with ST15 (RT010), which was normally considered as non-toxigenic in clade 1. However, ZR12 was found with positive *tcdA* and *tcdB*. Repeated experiments have been done to confirm the results. The reason for the isolates with the same RT and ST, but different toxin profile was unknown. RT023, with positive *tcdA, tcdB* and CDT, was assumed as the representative type in clade 3 (Bauer et al., 2011). Two isolates with ST 5 were found in clade 3 in this study, but the interesting thing is that none of them are RT023 (one is RT024, and the other one is un-identified). Distinct from the other 23 isolates with RT001 (*tcdA*^+^*tcdB*^+^*cdtA*^-^*cdtB*^-^), isolate 21074 (ST3, RT001, clade1) was *tcdA* negative. This may attributed to the variation of the DNA sequence of gene *tcdA* so primers used here are not specific for it.

With the exception of 23 non-toxigenic strains, the remaining *C*. *difficile* isolates were toxigenic, with *tcdA*^+^*tcdB*^+^*tcdC*^+^*tcdR*^+^*tcdE*^+^*cdtA*^-^*cdtB*^-^ the main toxin type identified. This observation is similar to previous reports in China and elsewhere around the world. Within the toxigenic isolates, there was one isolate that was negative for *tcdR*. Encoded by the *tcdR* gene, TcdR can positively regulate the expression of A/B toxin. When *tcdR* mutates, it may lead to a decrease in the toxin protein production and is likely to lead to a decrease in virulence of the bacteria (Antunes and Dupuy, 2010). Furthermore, there is another strain that was negative for the *tcdC* gene, which encodes the TcdC protein, a negative regulator of A/B toxin. When *tcdC* mutates, it can consequently lead to an increase in toxin production and is likely to lead to augmented bacteria virulence (Carter et al., 2011). In this experiment, 11 *C*. *difficile* isolates were found with binary toxin genes, of which one is RT027, six are RT078, one is RT024, and the rest three are RT unknown. Interestingly, two strains were only with positive for the *cdtA*^+^ gene but were negative *cdtB^-^* gene. These two patients are both with high risk factors, including age over than 85 years and hospitalized over than 1 month. Whether this organism was colonizing patients or was the cause for infection requires further confirmation.

By comparing the ST results, RT and toxin types of all *C. difficile* strains, there is not a consistent one-to-one match between ST and RT types. However, it was found that isolates with the same ST type had the same toxin type, with the exception of non-virulent strains. According to the whole genome MLST, six distinct phylogenetic clades (1-5 and C-I) were described (Janezic and Rupnik, 2015). In clades 1, 4, and 5, toxigenic strains were commonly combined with non-toxigenic strains. However, clade C-I is associated only with non-toxigenic strains (Dingle et al., 2014). In our study, all the isolates formed 5 clades from 1-5 without C-I, which has recently been described to include 5 STs, while all the non-toxingenic isolates were dispersed throughout clade1, 4, and 5. Clade 1 is the largest group containing diverse STs, which is consistent with clade 1 being the most heterogenous. In this study, the most predominant STs in China were from clade 1. Only one isolate was found as ST1 and this was confirmed to be hypervirulent RT 027, which is the representative of this ST. The most prevalent types in clade 3 were ST5, 22 and 25 (Stabler et al., 2012), with only two isolates (ST5) being discovered in clade 3 in China. Clade 4, known as the A-B+ clade, includes ST 37 (RT017) and another 15 additional STs (Knetsch et al., 2012). Following clade 1, clade 4 was the second largest cluster reported in this study, containing 31 isolates, which were ST37, 81, 39, 109, 332, all displaying distinct toxin profile (S1). Clade 5 includes ST11, with the most prominent representative RT being RT 078. RT 078 has emerged as a significant public health problem recently, whereas before it was most frequently associated with animals (Rupnik et al., 2008). Here, six out of 8 ST 11 isolates were confirmed as RT 078, which were all from older hospitalized patients in Beijing.

In summary, this is the first multi-center study of *C. difficile* isolates in China, which elucidates the molecular epidemiological characteristics of *C. difficile* in China across a large timespan. The toxin profile A+B+CDT- is the main type in China. Furthermore, ST35, ST3, ST37, and ST54 were identified as the predominant ST types in China, and RT 017, RT 001 and RT 012 were the most frequent types identified here. Nevertheless, one RT 027 from Hangzhou and 6 RT 078 isolates from Beijing were found, although no outbreaks were reported. In addition, we established a PCR-ribotyping library in China including ECDC-Brazier collection and ATCC isolates by capillary electrophoresis, which will help to further elucidate the epidemiology of CDI in China. This may also help in identifying hypervirulent RT 027 and RT 078 isolates with the same ST types.

## Author Contributions

X-sL and W-zZ performed the molecular typing of all isolates and drawing the pictures. X-sL wrote the draft manuscript. W-gL contributed to the culture and confirmed the tested isolates. J-xL reviewed the manuscript. YW conceived the study, supervised the research, and revised the manuscript.

## Conflict of Interest Statement

The authors declare that the research was conducted in the absence of any commercial or financial relationships that could be construed as a potential conflict of interest.

## Footnotes

1 https://pubmlst.org/cdifficile

## Supplementary Material

TABLE S1 Detailed results, including molecular types and toxin gene profiles of all 199 isolates.
